# FOXA1 Directs H3K4 Monomethylation at Enhancers via Recruitment of the Methyltransferase MLL3

**DOI:** 10.1016/j.celrep.2016.11.028

**Published:** 2016-12-06

**Authors:** Kamila M. Jozwik, Igor Chernukhin, Aurelien A. Serandour, Sankari Nagarajan, Jason S. Carroll

**Affiliations:** 1 Cancer Research UK Cambridge Institute, University of Cambridge, Robinson Way, Cambridge CB2 ORE, UK; 2 Genome Biology Unit, European Molecular Biology Laboratory, 69117 Heidelberg, Germany

**Keywords:** breast cancer, enhancers, H3K4me1, FOXA1, MLL3

## Abstract

FOXA1 is a pioneer factor that binds to enhancer regions that are enriched in H3K4 mono- and dimethylation (H3K4me1 and H3K4me2). We performed a FOXA1 rapid immunoprecipitation mass spectrometry of endogenous proteins (RIME) screen in ERα-positive MCF-7 breast cancer cells and found histone-lysine N-methyltransferase (MLL3) as the top FOXA1-interacting protein. MLL3 is typically thought to induce H3K4me3 at promoter regions, but recent findings suggest it may contribute to H3K4me1 deposition. We performed MLL3 chromatin immunoprecipitation sequencing (ChIP-seq) in breast cancer cells, and MLL3 was shown to occupy regions marked by FOXA1 occupancy and H3K4me1 and H3K4me2. MLL3 binding was dependent on FOXA1, indicating that FOXA1 recruits MLL3 to chromatin. MLL3 silencing decreased H3K4me1 at enhancer elements but had no appreciable impact on H3K4me3 at enhancer elements. We propose a mechanism whereby the pioneer factor FOXA1 recruits the chromatin modifier MLL3 to facilitate the deposition of H3K4me1 histone marks, subsequently demarcating active enhancer elements.

## Highlights

• Mass spectrometry of chromatin-associated proteins with FOXA1 identifies MLL3
• FOXA1 recruits MLL3 to deposit H3K4me1 on FOXA1-bound enhancers
• MLL3 promotes ERα-dependent gene transcription and proliferation
• GRHL2 co-occupies regions of ERα, FOXA1, and MLL3 on enhancers

## Graphical Abstract

**Figure:**
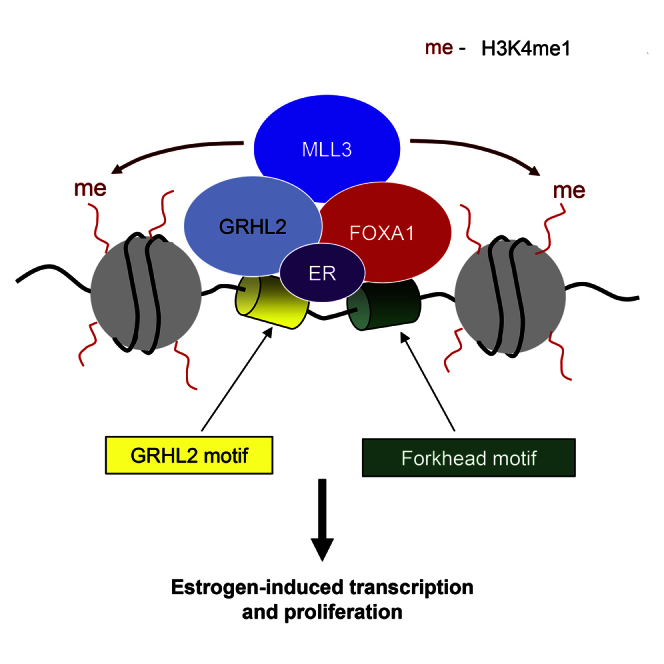

## Teaser

Jozwik et al. demonstrate that FOXA1 can activate enhancers by recruiting the chromatin-associated histone methyltransferase MLL3 to mediate monomethylation on enhancers. This identifies FOXA1 as an upstream regulatory element in the establishment of enhancers that regulates active chromatin marks.

## Introduction

FOXA1 (Forkhead box protein A1) is a pioneer factor (Jozwik and Carroll, 2012) that binds to condensed chromatin and allows subsequent binding of other transcription factors. FOXA1 contributes to chromatin opening to facilitate binding of estrogen receptor α (ER) in breast cancer (Carroll et al., 2005) and androgen receptor (AR) in prostate and breast cancer cells (Robinson et al., 2011, Sahu et al., 2011, Yang and Yu, 2015). ER is a driver of cell proliferation and tumor growth, and ER-positive breast cancer accounts for over 70% of all breast cancers (Curtis et al., 2012). Recent evidence has shown that FOXA1 is essential for almost all ER binding events in breast cancer (Hurtado et al., 2011) and for ER functionality, yet our understanding of FOXA1 activity and the events involved in determining FOXA1-chromatin interactions is limited.

FOXA1 binding occurs at enhancer regions enriched in histone 3 lysine 4 mono- and dimethylation (H3K4me1/me2) (Lupien et al., 2008). While it has been reported that FOXA1 binding requires H3K4me1/me2 marks (Lupien et al., 2008), more recent findings showed that exogenous expression of FOXA1 in the FOXA1-negative MDA-MB-231 cell line results in the acquisition of H3K4me1/me2 at FOXA1-bound sites (Sérandour et al., 2011), suggesting that FOXA1 may actually contribute to deposition of the H3K4me1 and H3K4me2 marks rather than associate with enhancers that are demarcated by the presence of these marks. Clearly, the order of these events is not resolved, yet FOXA1 binding and the H3K4me1/me2 signal result in a functional enhancer element that can recruit additional factors (such as ER) to drive expression of genes, including those involved in cell-cycle progression.

Unlike H3K4me1 and H3K4me2, which are typically found at enhancer elements, H3K4me3 is typically observed at promoter regions, and several investigations have associated the histone-lysine N-methyltransferase enzyme MLL3 with the deposition of H3K4me3 marks at promoters (Ardehali et al., 2011, Vermeulen and Timmers, 2010). More recently, the MLL3/MLL4 complex has been implicated in the regulation of H3K4me1 in mice (Herz et al., 2012). Importantly MLL3 is mutated in a number of solid cancers, including 8%–11% of breast cancers (Ellis et al., 2012, Wang et al., 2011), although a role for MLL3 in breast cancer and the functional consequences of these mutational events are not known. Silencing of MLL3 (and the related protein MLL2) has been shown to decrease the estrogen-mediated activation of HOXC6 in human placental choriocarcinoma (JAR cell line), and knockdown of either ERα or ERβ abolished estrogen-dependent recruitment of MLL2 and MLL3 onto the HOXC6 promoter in the JAR cell line (Ansari et al., 2011).

We sought to discover proteins that interact with FOXA1 in ER-positive (ER+) breast cancer cells by performing FOXA1 RIME (rapid immunoprecipitation mass spectrometry of endogenous proteins), an unbiased proteomic method that permits discovery of protein networks. This revealed a role for MLL3 as a critical chromatin regulatory protein at enhancer elements and as a factor that contributes to H3K4me1 deposition at these enhancers.

## Results

### RIME Analysis of FOXA1-Associated Proteins Reveals Interactions with MLL3 in Breast Cancer Cells

**Figure 1:**
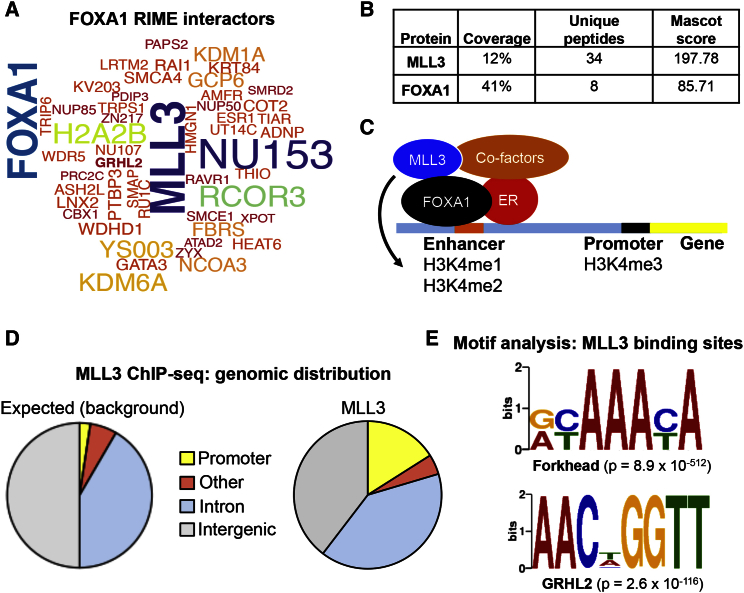
Purification of FOXA1-Associated Proteins Using RIME and Mapping of MLL3 Binding Genome-wide (A) The FOXA1 interactome was discovered by performing RIME in MCF-7 breast cancer cells. The data are represented as a Wordcloud, where the size of protein names represent the strength and confidence of the interactions based on the Mascot score. MLL3 was identified as one of the strongest and most reproducible FOXA1-interacting proteins. (B) Peptide coverage, number of unique peptides identified, and Mascot score of MLL3 and FOXA1 following FOXA1 purification. (C) Hypothesized mechanism of FOXA1 and MLL3 function. Our finding that MLL3 and FOXA1 physically interact in breast cancer cells implies that FOXA1 could recruit the enzyme that can add methyl groups to histone 3 lysine 4. FOXA1-bound enhancers are demarcated by H3K4me1 and H3K4me2. (D) MLL3 ChIP-seq was conducted and the genomic distribution of MLL3 peaks is shown relative to the whole genome (the expected control values). Regions bound by MLL3 occurred mostly at enhancers rather than promoters. (E) De novo motif analysis of MLL3 binding sites. Motif analysis revealed an enrichment in Forkhead motif, the canonical motif bound by FOXA1, and motifs for the transcription factor grainyhead-like 2 protein (GRHL2).

We performed RIME (Mohammed et al., 2013) of FOXA1 in MCF-7 breast cancer cells to identify endogenous FOXA1 interactors. Asynchronous MCF-7 cells were grown in full media, and five replicates of FOXA1 RIME were conducted. MLL3 was identified as one of the strongest and most reproducible interactors (Figure 1A), and the peptide coverage, number of unique peptides identified, and Mascot score of MLL3 and FOXA1 are shown in Figure 1B. A full list of FOXA1-interacting proteins is provided in Table S1. We hypothesized that this interaction may be functional, as the pioneer factor FOXA1 binds at enhancer regions enriched in H3K4me1/me2 histone marks and FOXA1 has been shown to contribute to the acquisition of the H3K4me1/me2 mark (Sérandour et al., 2011). In addition, loss of MLL3 has been previously shown to correlate with reduced H3K4me1 at specific regions within the genome in mice (Herz et al., 2012). Our findings that MLL3 and FOXA1 physically interact in breast cancer cells implies that FOXA1 may be able to directly recruit the enzyme that can add methyl groups to the H3K4 residue (Figure 1C).

### Global Mapping of MLL3 Binding Sites Shows Enrichment at Enhancers

Due to the size of MLL3 (∼540 kDa) and the fact that FOXA1 is the same size at the antibody heavy chain, we were unable to conduct co-immunoprecipitation (co-IP) validation experiments on the MLL3-FOXA1 interaction. However, we explored this putative interaction in several ways. We reversed the pull-down, purified MLL3, and could discover FOXA1 as an interacting protein by RIME (data not shown). In addition, we assessed the global interplay between MLL3 and FOXA1 binding events by performing MLL3 and FOXA1 chromatin immunoprecipitation sequencing (ChIP-seq). Asynchronous MCF-7 cells were grown and triplicate ChIP-seq experiments were conducted. MLL3 ChIP-seq was conducted using a custom antibody that we validated by RIME and could show to be specific to MLL3 (Figure S1A). Data from all the replicates were pooled, and peaks were called using model based analysis for ChIP-seq (MACS) (Zhang et al., 2008), resulting in a total of 10,772 MLL3 binding events in MCF-7 cells. MLL3 has been previously implicated as an enzyme that contributes to H3K4me3 deposition at promoter regions (Ananthanarayanan et al., 2011), but our ChIP-seq data showed that MLL3 binding was mostly distributed at enhancer elements and intergenic regions (Figure 1D) with a smaller fraction distributed at promoters, similar to what has been observed for ER and FOXA1 (Carroll et al., 2005, Hurtado et al., 2011). Analysis of enriched DNA motifs within the MLL3 binding sites revealed an enrichment of Forkhead motifs, the canonical motif bound by FOXA1 (MEME e-value = 8.9e-512). In addition, motifs for the transcription factor grainyhead-like 2 protein (GRHL2) were identified (Figure 1E) (MEME e-value = 2.6e-116), although there is limited information linking GRHL2 and ER/FOXA1 signaling.

### MLL3 Binding Is Dependent on FOXA1 and Is Required for ER Activity

**Figure 2:**
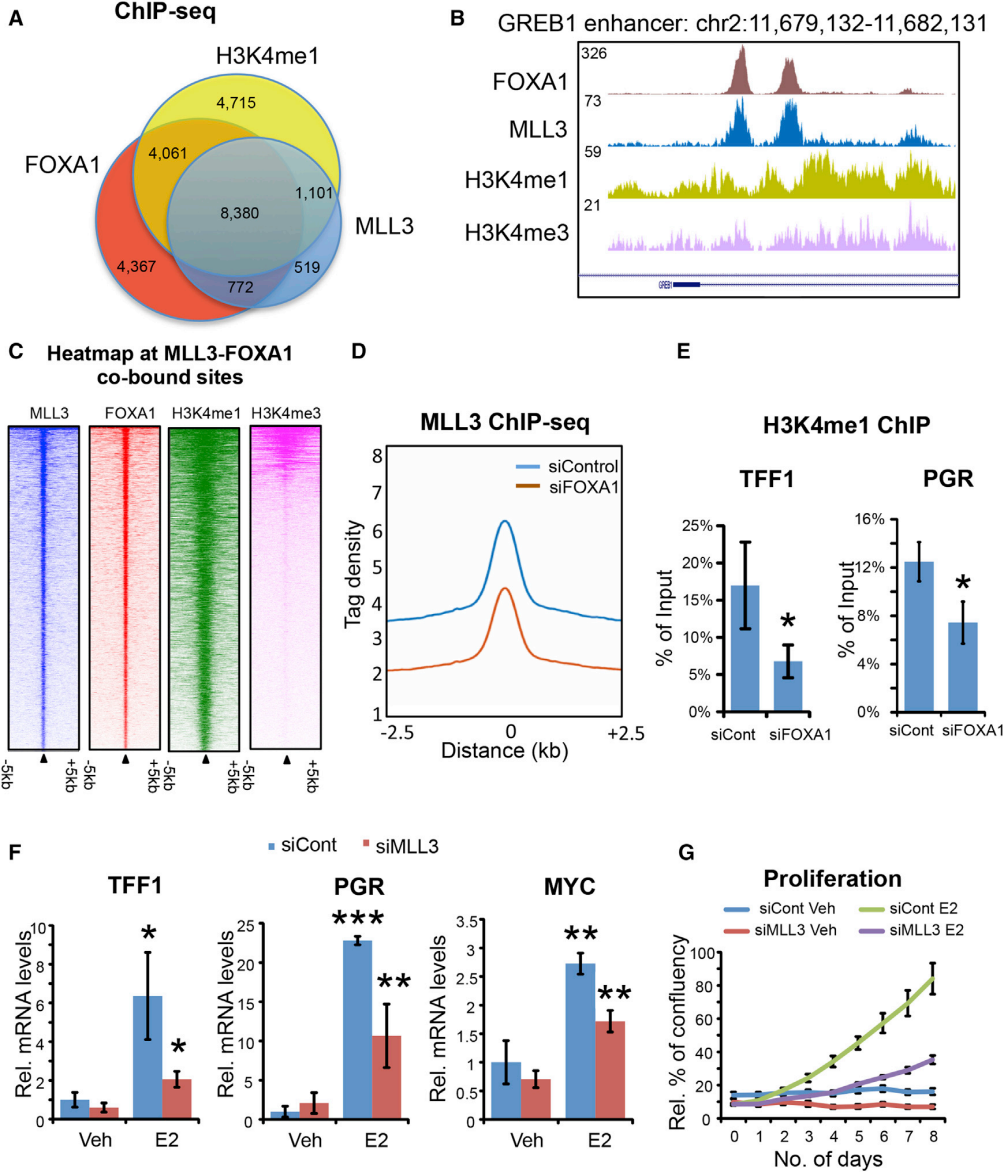
Co-binding of MLL3, FOXA1, and H3K4me1/me3 and Mechanism of MLL3 Recruitment (A) Overlap of MLL3, FOXA1, and H3K4me1 binding revealed by ChIP-seq. MLL3 binding sites were co-bound by FOXA1 and the histone marks. The numbers of peaks within each category are shown on the diagram. (B) An example of an MLL3, FOXA1, and H3K4me1/me3 co-bound region at the GREB1 enhancer. (C) Heatmap of MLL3-FOXA1 co-bound regions showing binding signal intensity for FOXA1, MLL3, H3K4me1, and H3K4me3. Binding is ranked from the strongest to the weakest binding sites. (D) Signal intensity plot representing changes in MLL3 ChIP-seq signal in siControl versus siFOXA1-transfected conditions. Differentially bound sites needed to be detected in at least two replicates to be included. (E) ChIP-qPCR analyses of H3K4me1 after knockdown of FOXA1 on ER-bound enhancers of *TFF1* and *PGR*. n = 3; mean ± SD is shown as the of percentage of input. ^∗^p ≤ 0.05. (F) qRT-PCR of estrogen-induced genes *TFF1* and *PGR* with or without knockdown of MLL3 after 3 days of charcoal-stripped serum ±10 nM estrogen (E2) treatment. n = 3; mean ± SD is shown in average relative mRNA levels compared to the vehicle (Veh) condition. ∗p < 0.05, ∗∗p < 0.01, ∗∗∗p < 0.001. (G) Estrogen-induced proliferation assays with or without knockdown of MLL3 after 3 days of charcoal-stripped serum ±10 nM estrogen treatment for 8 days. n = 4; mean ± SEM of percentage of confluency is shown.

ChIP-seq of FOXA1 and H3K4me1/me3 were performed in asynchronous MCF-7 cells in triplicate, and peaks were called using MACS, revealing 23,375 FOXA1 peaks, 26,584 H3K4me1, and 13,478 H3K4me3 peaks. The binding of FOXA1 and H3K4me1/me3 was overlapped with the MLL3 binding sites. The majority (55.8%) of MLL3 binding events were co-bound by FOXA1 (Figure 2A), and since H3K4me1 is associated with FOXA1, it was not unexpected that MLL3/FOXA1-co-bound regions were also typically marked by H3K4me1 (Figure 2A). A small percentage (9.1%) of MLL3/FOXA1 co-bound regions were also marked by histone 3 lysine 4 trimethylation (H3K4me3) (Figure S2B). An example of a MLL3 and FOXA1 co-bound region, marked by both H3K4me1 and H3K4me3, is shown in Figure 2B. Heatmap visualization of the FOXA1 binding and H3K4me1/me3 signal at the MLL3 binding events is shown in Figure 2C, indicating that a substantial degree of the MLL3 and FOXA1 co-bound regions also possess H3K4me1 signal.

Given that MLL3 was the top FOXA1-interacting protein (Figure 1A), that MLL3 binding sites were enriched for Forkhead motifs, and that 55.8% of MLL3 binding events we also FOXA1 binding sites, we hypothesized that MLL3 was recruited to the chromatin by FOXA1. To assess this, MCF-7 cells were transfected with small interfering RNA (siRNA) to control (siControl) or siRNA to FOXA1, and effective FOXA1 silencing was confirmed. Following FOXA1 silencing, MLL3 ChIP-seq was conducted in triplicate independent biological replicates. MLL3 peaks were called in siControl or siFOXA1-transfected conditions. This resulted in a global decrease in MLL3 binding when FOXA1 was depleted (Figure 2D). The decreased MLL3 binding following silencing of FOXA1 was not due to a decrease in MLL3 expression, since MLL3 mRNA levels increased following FOXA1 silencing (Figure S2A). Six MLL3 binding sites were assessed using ChIP-qPCR, validating the dependence on FOXA1 for MLL3 binding to chromatin (Figure S2C). Given the importance of FOXA1 in recruiting MLL3 to the chromatin, we speculated that FOXA1 promotes H3K4me1 to activate enhancers via MLL3. Consistently, knockdown of FOXA1 decreased H3K4me1 as well as H3K27ac on ER-dependent enhancers (as shown in Figure S2D), which are absent for H3K27me3 (Figures 2E and S2E). Importantly, MLL3 knockdown showed a significant decrease in ER-induced gene transcription and proliferation (Figures 2F, 2G, and S2G), which highlights the importance of MLL3 in ER-activated transcription.

### Chromatin Properties at MLL3 Binding Events

**Figure 3:**
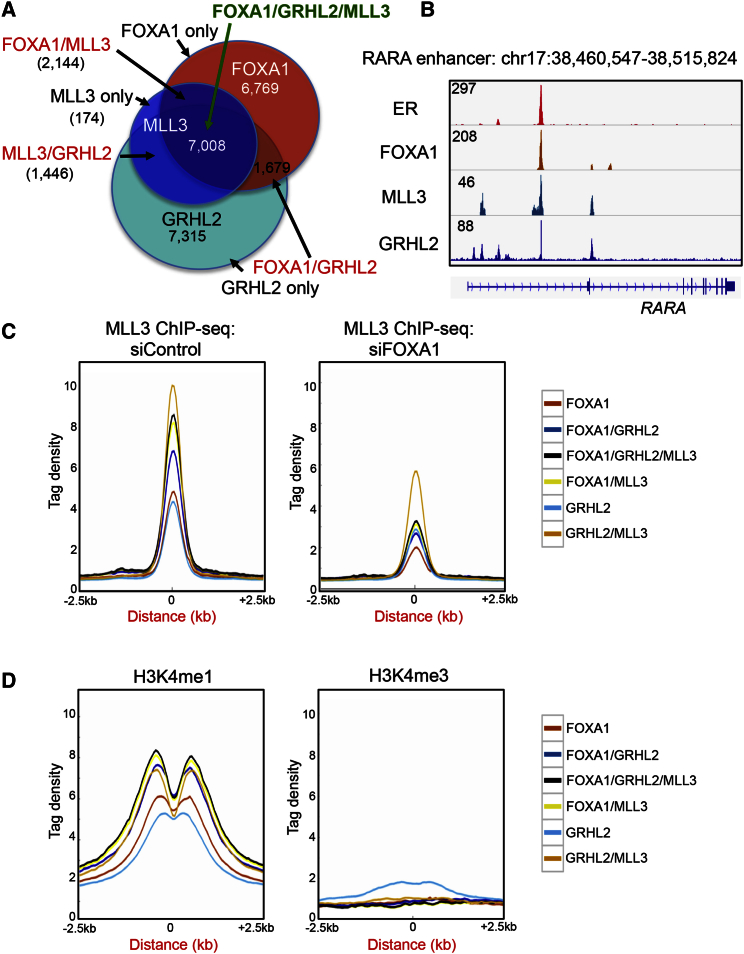
Functional Distinction between Regions Bound by FOXA1, GRHL2, and MLL3 (A) Venn diagram showing the overlap of MLL3, FOXA1, and GRHL2 binding regions, identifying the different categories of binding events. For subsequent analyses, we assessed the number of regions co-bound by one factor (FOXA1 only, MLL3 only, or GRHL2 only), two factors (FOXA1 and MLL3, MLL3 and GRHL2, or GRHL2 and FOXA1), and all three factors. (B) An example of an MLL3, FOXA1, ERα, and GRHL2 co-bound region. (C) Average MLL3 binding signal in siControl and siFOXA1 conditions at the different binding categories. Following FOXA1 silencing, MLL3 binding intensity was reduced at regions occupied by MLL3, FOXA1, and GRHL2, regions occupied by MLL3 and FOXA1, and to a lesser extent at regions occupied by MLL3 and GRHL2. (D) H3K4me1/me3 distribution at the different binding regions. The most enriched H3K4me1 regions were those where MLL3 was recruited.

As previously observed (Figure 1E), GRHL2 (grainyhead-like 2 protein) motifs were enriched within MLL3 binding events. GRHL2 was also found to be a FOXA1 interacting protein from the RIME experiments (Figure 1A), suggesting that the enrichment of GRHL2 motifs might represent a functional interaction between FOXA1 and GRHL2. The role of GRHL2 in breast cancer is currently unclear, with both pro-metastatic and anti-metastatic roles (Werner et al., 2013, Xiang et al., 2012). We performed GRHL2 ChIP-seq in MCF-7 cells in triplicate, and GRHL2 peaks were called using MACS, revealing 30,143 GRHL2 binding sites. GRHL2 binding was overlaid with MLL3 and FOXA1 binding, revealing 5,585 regions that were occupied by all three factors with significant overlap with ERα (Figures 3A and S3). An example of a co-occupied site is shown in Figure 3B. In total, 91.5% of MLL3 binding sites were co-occupied by FOXA1 and/or GRHL2. To gain insight into the mechanisms involved in the different *cis*-regulatory elements, we explored the seven different categories of binding by investigating regions bound by a single factor (FOXA1 only, MLL3 only, or GRHL2 only), two factors (FOXA1 and MLL3, MLL3 and GRHL2, or GRHL2 and FOXA1), or all three factors and used them for further analyses. Only 1.6% of the MLL3 binding regions were not co-bound by FOXA1, GRHL2, or both, suggesting that MLL3 cannot associate with chromatin without one of the associated transcription factors, and the MLL3-only binding regions were subsequently eliminated from further analyses.

In control conditions, MLL3 binding was most enriched at sites co-occupied by FOXA1, GRHL2, or both proteins together, suggesting that optimal MLL3-chromatin occupancy involves at least one of the additional transcription factors (Figure 3C). Following silencing of FOXA1, MLL3 binding was substantially reduced at two categories: the first was the regions bound by all three proteins, and the second was the FOXA1 and MLL3 (but not GRHL2) regions. Interestingly, MLL3 binding signal at MLL3 and GRHL2 (but not FOXA1) occupied *cis*-regulatory elements were moderately affected by FOXA1 silencing, suggesting multiple modes of MLL3-chromatin occupancy (Figure 3C). This suggests that upon FOXA1 silencing, MLL3 binding sites were lost at any region where FOXA1 co-binds, even if GRHL2 is also present, but MLL3 binding is moderately affected at regions where GRHL2 is the sole protein associated with MLL3.

When the different MLL3 binding regions were integrated with the H3K4me1/me3 data, the most enriched regions were those where MLL3, FOXA1, and GRHL2 were co-bound and those where MLL3 and FOXA1 were co-bound, although any region occupied by MLL3 had an increased H3K4me1 signal relative to regions occupied by FOXA1 or GRHL2, but not MLL3 (Figure 3D). These findings confirm that the presence of MLL3 correlates with increased H3K4me1.

Since FOXA1 contributes to the establishment of enhancer elements that are subsequently used by transcription factors such as ER in these breast cancer cells, we integrated the MLL3, FOXA1, and GRHL2 ChIP-seq data with ER binding information. As expected (Figures S2D), the regions bound by FOXA1 and MLL3 are commonly co-occupied by ER, in support of their role in establishing ER enhancer elements.

### H3K4me1 at Enhancer Elements Is Dependent on MLL3

**Figure 4:**
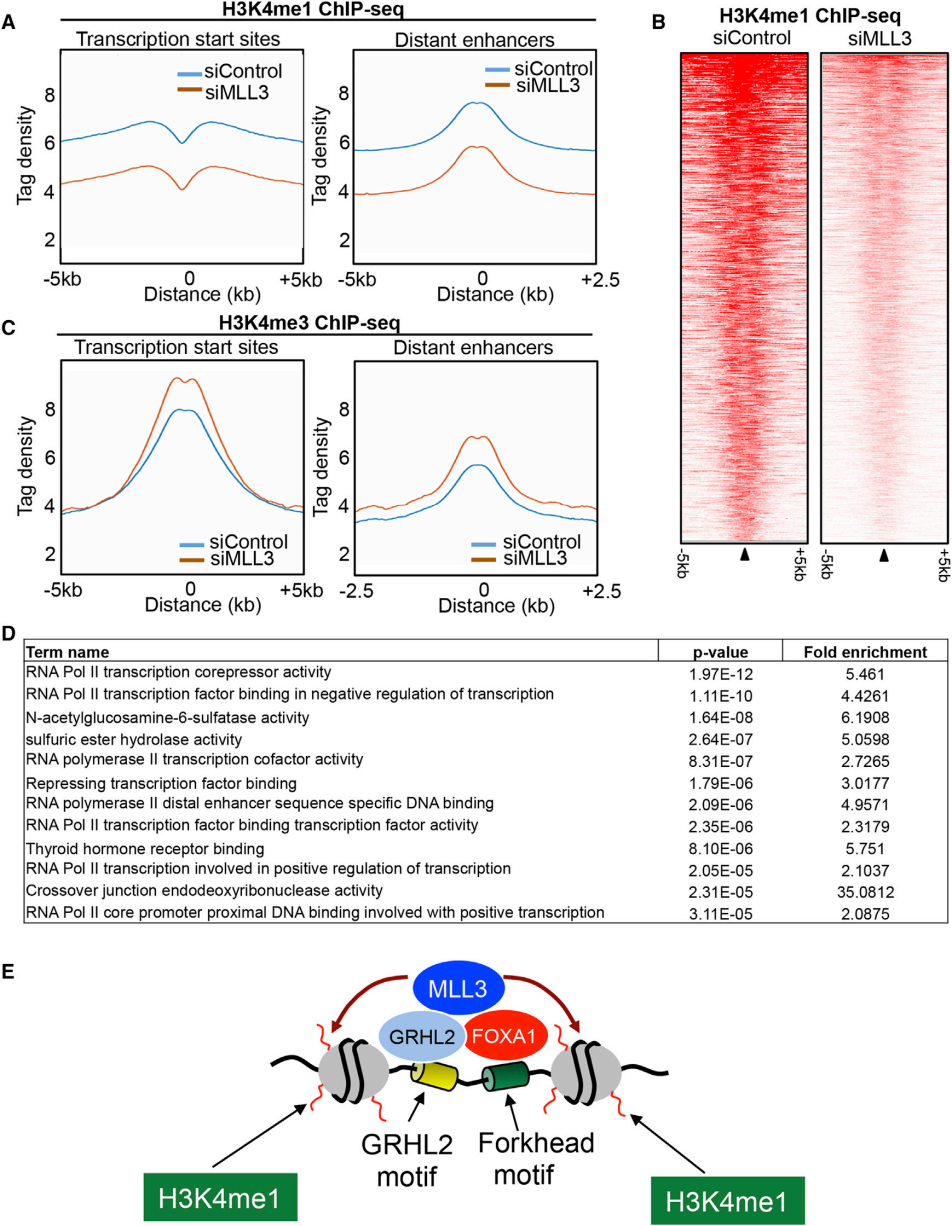
Direct Dependency of H3K4me1 on MLL3 at Enhancers The effect of MLL3 silencing on H3K4me1 and H3K4me3 binding were assessed by ChIP-seq in siControl or siMLL3-transfected cells. Differential H3K4me1 and H3K4me3 peaks that were altered by silencing of MLL3 were identified. (A) The average signal intensity of H3K4me1 at enhancer elements or promoters following silencing of MLL3. (B) Heatmap of differential H3K4me1 regions that occur at FOXA1/MLL3 co-bound regions. (C) The average signal intensity of H3K4me3 at enhancer elements or promoters following silencing of MLL3. (D) Enriched pathways within the 776 FOXA1/MLL3 co-bound regions that had a decreased H3K4me1 signal following silencing of MLL3. (E) Model showing FOXA1 and GRHL2 recruitment of MLL3, which subsequently contributes to monomethylation of H3K4.

Given that MLL3 binding was associated with regions enriched in H3K4me1 marks and MLL3 is a methyltransferase, we hypothesized that MLL3 contributes to the presence of this methyl mark at enhancer elements. To assess this, MCF-7 cells were transfected with siControl or siMLL3 and triplicate H3K4me1 and H3K4me3 ChIP-seq experiments were conducted, and peaks were called using MACS. When MLL3 was silenced, deposition of H3K4me1 was substantially decreased at both enhancer elements and promoters (Figure 4A). We specifically assessed the changes in H3K4me1 at regions bound by both FOXA1 and MLL3, resulting in the identification of 776 FOXA1/MLL3-bound enhancers that had decreased H3K4me1 following MLL3 silencing (Figure 4B). There was no decrease in H3K4me3 at either the enhancer elements or the promoter regions when MLL3 was silenced and a modest gain of signal at both promoters and enhancer elements were observed (Figure 4C). We assessed the 776 enhancer elements with decreased H3K4me1 following MLL3 silencing for enriched pathways. Genomic Regions Enrichment of Annotations Tool (GREAT) revealed a number of enriched pathways, most of which were associated with transcriptional regulation (Figure 4D). Given the observation that regions bound by MLL3 possess the highest H3K4me1 signal (Figure 3D) and that H3K4me1 was depleted at enhancers when MLL3 was silenced, we postulate that H3K4me1 deposition is mediated by MLL3 at enhancer elements, as determined by FOXA1 and/or GRHL2 recruitment of MLL3. In support of this, MCF-7 cells were transfected with siControl or siFOXA1, and H3K4me1 ChIP-qPCR was conducted on a select number of loci (the genomic regions and the relative factor binding is shown in Figure S2H). Following inhibition of FOXA1, the H3K4me1 signal was diminished at some of the assessed loci (Figures 2E and S2F), and H3K27Ac was also decreased following FOXA1 inhibition (Figure S2E), which implies that FOXA1 and MLL3 are required for transcriptional activity from enhancer elements.

## Discussion

We propose a mechanism whereby the pioneer factor FOXA1 interacts with chromatin and recruits the methyltransferase MLL3, facilitating the deposition of H3K4me1 in breast cancer cells (Figure 4E). This denotes that an enhancer-specific pioneer factor FOXA1 can interact with a chromatin modifier (MLL3) to facilitate the occurrence of the H3K4me1 histone mark at regions that become functional enhancer elements. We have described links among FOXA1, MLL3, and H3K4methylation, revealed by RIME, an unbiased proteomic technique that showed MLL3 to be a robust FOXA1-interacting protein in MCF-7 breast cancer cells. MLL3 has been shown to contribute to H3K4me3 at promoter regions (Ananthanarayanan et al., 2011), but our evidence would suggest that MLL3 can also contribute to H3K4me1 marks at enhancer regions and that this is determined by the transcription factors that recruit MLL3 to the chromatin. Two mechanisms for MLL3 recruitment to chromatin are revealed by mining of MLL3 ChIP-seq data. Motifs for Forkhead and GRHL2 transcription factors were identified. Interestingly, MLL3-chromatin occupancy was shown to occur via FOXA1, GRHL2, or both factors, and our functional experiments confirm that FOXA1 is essential for MLL3 binding. GRHL2 has been implicated in metastasis in breast cancer (Werner et al., 2013, Xiang et al., 2012), and we hypothesize that its influence on cell migration and metastatic potential is attributed to its ability to tether MLL3 at chromatin and mediate enhancer activity. Whether GRHL2 is involved in ER/FOXA1+ breast cancer or can function independently of FOXA1 (and ER) is not clear, but GRHL2 is located on chromosome 8 and is commonly co-amplified with c-Myc, suggesting that any role for GRHL2 in mediating recruitment of the enzyme MLL3 may be substantially altered in tumors with the commonly occurring chromosome 8 amplification.

The predominant paradigm is that H3K4me1 and H3K4me2 marks are signatures of enhancer regions, whereas H3K4me3 is enriched at the promoters of coding genes (Calo and Wysocka, 2013, Heintzman et al., 2007, Heintzman et al., 2009). Our findings would suggest that the H3K4me1 marks at enhancers are enriched at FOXA1-bound enhancer elements, because this pioneer factor is able to recruit the enzyme that contributes to the deposition of this methylation event at regions co-bound by FOXA1, GRHL2, and the methyltransferase MLL3. Recently, it has been reported that MLL3/4 contributes to monomethylation (H3K4me1) of promoter regions in myoblasts (Cheng et al., 2014). It has also been shown that Trr, the *Drosophila* homolog of the mammalian MLL3/4 COMPASS-like complexes, can function as a major H3K4 monomethyltransferase on enhancers in vivo (Herz et al., 2012), with a modest decrease of H3K4me1 in mouse embryonic fibroblasts (MEFs) from MLL3 knockout mice (Herz et al., 2012). In our breast cancer cells, we see a pronounced depletion of H3K4me1 following MLL3 silencing. Since MLL3 and the related protein MLL4 function as a complex, it is possible that both MLL3 and MLL4 contribute to the enhancer H3K4 methylation marks, as both proteins needed to be deleted in MEFs in order to produce the decrease in the H3K4me1 (Herz et al., 2012). However, we did not find MLL4 as a FOXA1-interacting protein, and no FOXA1-MLL4 interactions were observed, even in MLL3-depleted cells (data not shown), suggesting a lack of redundancy between MLL3 and MLL4 in our breast cancer cells. It has also been shown that unlike MLL3, the depletion of MLL4 had no effect on the estrogen-mediated activation of HOXC6 (Ansari et al., 2011), suggesting that MLL4 is not functionally linked with ER biology. The specific role for MLL3 in ER+ breast cancer is supported by the recent finding that MLL3 was mutated in 5 out of 46 ER+ breast cancer samples (Ellis et al., 2012), and although the mutations occur at distinct regions within MLL3, a common result is putative pertubation in key enzymatic domains within MLL3. The functional significance of these mutations warrants investigation, although the large size of MLL3 (541 kDa) makes these functional experiments a challenging endeavor.

Taken together, we propose a mechanism by which the pioneer factor FOXA1 interacts with the chromatin modifier MLL3 to facilitate monomethylation of H3K4 at enhancer elements, resulting in the potential for transcription from these enhancer regions. These findings imply that the transcription factors that associate with enhancer elements are capable of actively contributing to the H3K4me1 that occurs at enhancers rather than requiring H3K4me1 presence for chromatin occupancy.

## Experimental Procedures

### Cell Lines

MCF-7 cells were obtained from ATCC. MCF-7 cells were grown in DMEM supplemented with 10% heat-inactivated fetal bovine serum (FBS), 2 mM L-glutamine, 50 U/mL penicillin, and 50 μg/mL streptomycin. All cell lines were regularly genotyped using STR profiling (Promega GenePrint 10 system). Cell lines were regularly tested for mycoplasma infection.

### Antibodies

The antibodies used for ChIP-seq were anti-FOXA1 (ab5089) from Abcam, anti-H3K4me1 (ab8895) from Abcam, anti-H3K4me3 (05-1339) from Millipore, anti-H3K27ac (C15410196) from Diagenode, anti-H3K27me3 (C15410195) from Diagenode, anti-GRHL2 (HPA004820) from Sigma-Aldrich, and negative control immunoglobulin G (IgG) anti-rabbit IgG (sc-2027), and anti-goat (sc-2028) from Santa Cruz Biotechnology. The custom anti-MLL3 antibody was provided by Prof. Ali Shilatifard (Stowers Institute, Kansas City, MO, and Northwestern University Feinberg School of Medicine, Chicago, IL).

### ChIP-Seq

ChIP was performed as described previously (Schmidt et al., 2009). ChIP-seq and the input libraries were prepared using the TruSeq ChIP Sample Prep Kit (Illumina). ChIP-seq of each factor was performed in at least biological triplicates. Reads were mapped to hg19 genome using Gsnap version 2015-09-29 (Wu and Nacu, 2010). Aligned reads with the mapping quality less than five were filtered out. The read alignments from three replicates were combined into a single library, and peaks were called with model based analysis for ChIP-seq 2 (MACS2) version 2.0.10.20131216 (Zhang et al., 2008) using sequences from MCF-7 chromatin extracts as a background input control. For the ChIP samples of histones with mono- and trimethyl modifications, the broad peaks were called. The peaks yielded with MACS2 q value ≤ 1e-5 were selected for downstream analysis. Meme version 4.9.1 (Bailey et al., 2009) was used to detect known and discover binding motifs among tag-enriched sequences. For visualizing tag density and signal distribution heatmap, the normalized tumor read coverage in a window of a ±2.5- or 5-kb region flanking the tag midpoint was generated using a bin size of 1/100 of the window length. Differential binding analysis (Diffbind) was performed as described previously (Stark and Brown, 2011).

For ChIP-qPCR, primer sequences used were TFF1 forward, 5′-GTGGTTCACTCCCCTGTGTC-3′; TFF1 reverse, 5′-GAGGCATGGTACAGGAGAGC-3′; GREB1 forward, 5′-CACGTCCCCACCTCACTG-3′; GREB1 reverse, 5′-TGTTCAGCTTCGGGACACC, PGR forward, 5′-GCTCCAGCTAACTGATGGTCTG-3′; PGR reverse, 5′-TGGGCCTAGATTATTGAGTTCAGG-3′.

### RIME

RIME was performed as previously described (Mohammed et al., 2013). Proteins were digested using trypsin. Maximum allowed missed cleavage was 2, the peptide threshold was 95% and the protein false discovery rate (FDR) was set to 0.5%. Proteins were considered as interactors when at least 2 high-confident peptides were identified and when none of these peptides were observed in matched IgG control RIME experiments. Additionally, FOXA1 interactors were filtered using the CRAPome database (http://www.crapome.org).

### Small Interfering RNA Transfections

siRNAs used to silence FOXA1 were obtained from Dharmacon RNAi Technologies. The sequence of the siRNA that targeted FoxA1 is 5′-GAGAGAAAAAAUCAACAGC-3′ and has been previously validated (Hurtado et al., 2011). Small interfering Smartpool RNAs used to silence MLL3 were obtained from Dharmacon (L-007039-00-0020 and MQ-004828-02-0002). AllStars Negative Control siRNA (QIAGEN) and siGenome Non-targeting siRNA (D-001210-02-05) from Dharmacon were used as a negative controls. Cells were transfected with siRNA using Lipofectamine 2000 (Invitrogen) and Lipofectamine RNAiMAX (Thermo Scientific).

### Preparation of mRNA

Cells cultured in 15-cm^2^ dishes were first washed twice with cold PBS, and RNA was extracted using the RNeasy kit (QIAGEN) per the manufacturer’s instructions. DNA was degraded by adding 20 U RNase-free DNaseI (Roche Diagnostics GmbH) for 15 min at room temperature. DNase I treatment was performed on columns.

### Preparation of cDNA

200 ng to 1 μg total RNA was diluted to a final volume of 11 μL using 100 μg random primers (Promega), 2.5 mM dNTP mix, and nuclease-free water. This mixture was then incubated at 65°C for 5 min. First-strand buffer (Invitrogen) and 10 mM 1.4 DTT (Invitrogen) was then added, and this mixture was incubated at 25°C for 10 min to allow primer annealing. The mixture was then heated at 42°C for 1 min, and 200 U SuperScript III Reverse Transcriptase (Invitrogen) was added. The final mixture was then incubated at 42°C for an additional 50 min, and the process was stopped after inactivating the enzyme at 70°C for 15 min. The resulting cDNA was then diluted 1:10 in H_2_O for subsequent use.

### qRT-PCR

qPCR was performed using a Stratagene Mx3005P RealTime machine. Each qPCR reaction contained Power SYBR green PCR Master Mix (Applied Biosystems), 250 nM of each primer, 2 μL DNA eluted after chromatin immunoprecipitation, and nuclease-free H_2_O added to a final volume of 20 μL. The PCR program consisted of first heat-activating the Taq polymerase at 95°C for 10 min. This was then followed by 45 cycles of 15 s at 95°C and 30 s at 60°C. The fluorescence from each well was analyzed at every cycle. The final step involved increasing the temperature from 65°C to 95°C and continuously reading the fluorescence. Reactions were performed in triplicate, and results were analyzed using the delta-delta Ct method (Livak and Schmittgen, 2001). The enrichment was normalized with control mRNA levels of ubiquitin C (UBC), and relative mRNA levels were calculated comparing to vehicle.

For qRT-PCR, primers used were UBC forward, 5′-ATTTGGGTCGCGGTTCTTG-3′; UBC reverse, 5′-TGCCTTGACATTCTCGATGGT-3′; TFF1 forward, 5′-GTGTCACGCCCTCCCAGT-3′; TFF1 reverse, 5′-GGACCCCACGAACGGTG-3′; PGR forward, 5′-CTTAATCAACTAGGCGAGAG-3′; PGR reverse, 5′-AAGCTCATCCAAGAATACTG-3′; MYC forward, 5′-GCCACGTCTCCACACATCAG-3′; MYC reverse, 5′-TCTTGGCAGCAGGATAGTCCT-3′; GREB1 forward, 5′-GCTAACCATGCTGCAAATGA-3′; GREB1 reverse, 5′-ACACAGTCAGGGCAAAGGAC-3′; MLL3 forward, 5′-TGCCTGTTCTCAGTGTGGTC-3′; MLL3 reverse, 5′-TCACACAGCAGGAGTCTTCC; FOXA1 forward: 5′-GGGGGTTTGTCTGGCATAGC-3′; and FOXA1 reverse, 5′-GCACTGGGGGAAAGGTTGTG-3′.

### Proliferation Assays

Proliferation assays were performed in Incucyte analysis system (FLR 10X from Essen Bioscience). After reverse transfection with siRNAs, cells were treated with charcoal stripped serum (kindly provided by Dr. Mohammed Asim, CRUK, Cambridge, UK) for 3 days and then treated with 10 nM estrogen (Sigma), and cell confluence was assessed. Relative confluency was calculated by comparing it to that of estrogen-treated control siRNA conditions.

### Statistical Methods

To detect significant regions bound with each factor from ChIP-seq data during MACS2, the threshold of q value ≤ 1e-5 was used. FDR ≤ 0.5% was used for RIME data analysis. The e-value was used to detect significant motifs from MEME analysis. For qPCR analyses, p values were calculated using ANOVA or Student’s t test, and values ≤ 0.05 were considered as significant. The bar graphs were represented as mean ± SD for qPCR and mean ± SEM for proliferation assays. Three to four biological replicates were used throughout the study.

## Author Contributions

K.M.J., S.N, and J.S.C. designed experiments. K.M.J., S.N., and A.A.S. performed experiments and analyzed the data, and I.C. analyzed the genomic and proteomic data. K.M.J., S.N., and J.S.C. wrote the manuscript, with input from all authors. J.S.C. oversaw the work.

## Accession Numbers

The accession numbers for the ChIP-seq and proteomic data reported in this paper are GEO: GSE81714 and PRIDE: PXD005253, respectively.

## Supplemental Information

Document S1. Figures S1–S3

Table S1. Table of FOXA1 Interacting Proteins Identified by RIME

Document S2. Article plus Supplemental Information
