# Supplementary material for: FOXA1 Directs H3K4 Monomethylation at Enhancers via Recruitment of the Methyltransferase MLL3
Source: Cell Rep. 2016 Dec 6;17(10):2715–23. doi: 10.1016/j.celrep.2016.11.028 (PMC5177601; doi:10.1016/j.celrep.2016.11.028)

Cell Reports, Volume *17*

Supplemental Information

FOXA1 Directs H3K4 Monomethylation at Enhancers via Recruitment of the Methyltransferase MLL3

Kamila M. Jozwik, Igor Chernukhin, Aurelien A. Serandour, Sankari Nagarajan, and Jason S. Carroll

Supplementary Figure 1A. **Validation of MLL3 antibody using RIME. A.** Coverage of MLL3 protein in Mass Spectrometry. All regions of MLL3 protein were represented in Mass spectrometry. **B.** Mass spectrometry spectrum of the MLL3 protein. MLL3 was the top ranked protein following purification using the MLL3 antibody.

A

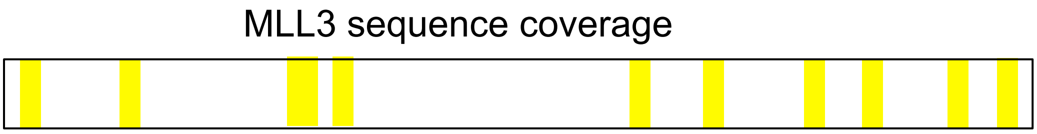

B

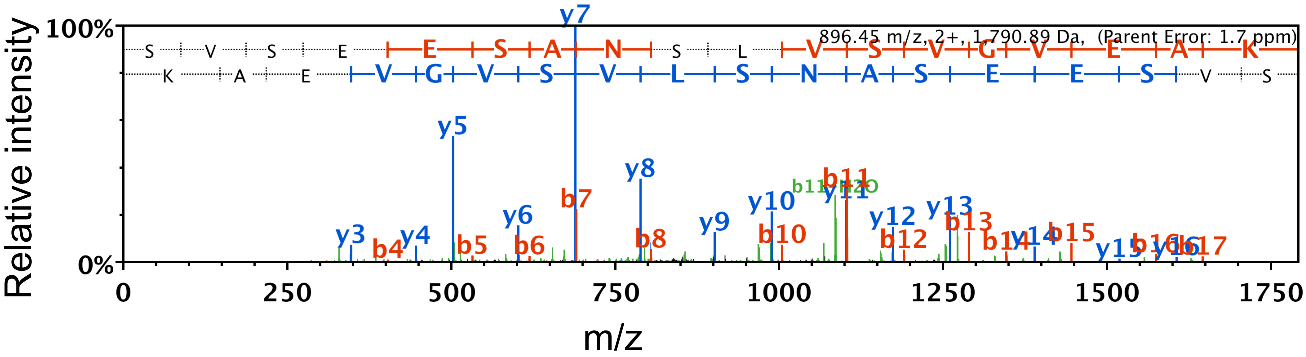

Supplementary Figure 2. **A**. Confirmation of effective FOXA1 silencing. FOXA1 expression upon silencing of FOXA1, confirming knockdown. siCt = siControl. Also included is mRNA changes in MLL3 after silencing of FOXA1. * denotes $p < 0.05$, using T-test. **B.** Overlap between MLL3, FOXA1, H3K4me1 and H3K4me3 binding revealed by ChIP-seq. MLL3 binding sites were co-bound by FOXA1 and the histone marks. The numbers of peaks within each category is shown.

A

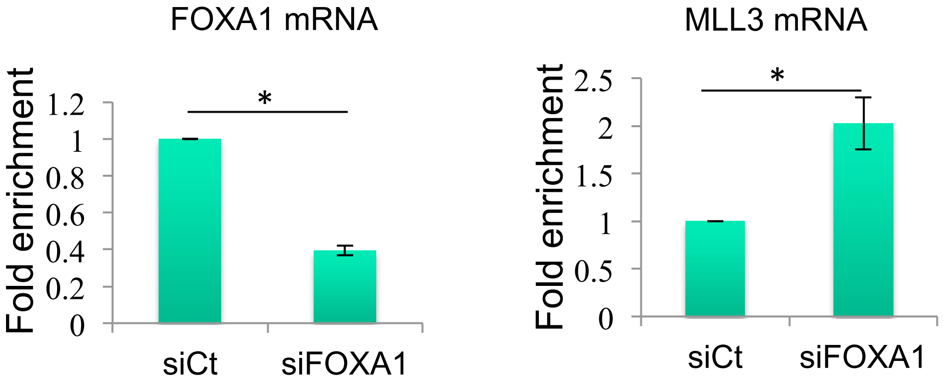

B

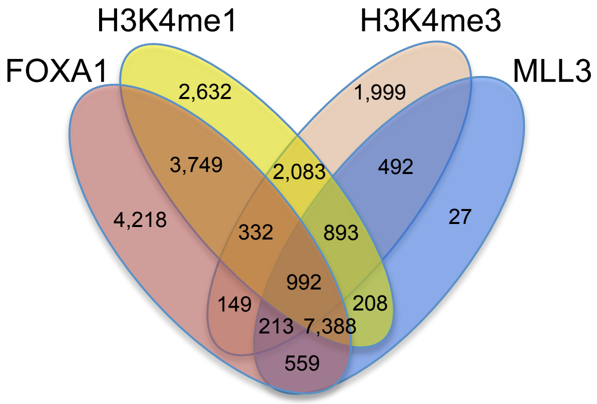

Supplementary Figure 2C. ChIP-qPCR validation of MLL3 binding sites that were lost upon FOXA1 silencing. The ChIP data is shown as relative change to siControl. * denotes $p < 0.05$, as determined by t-test.

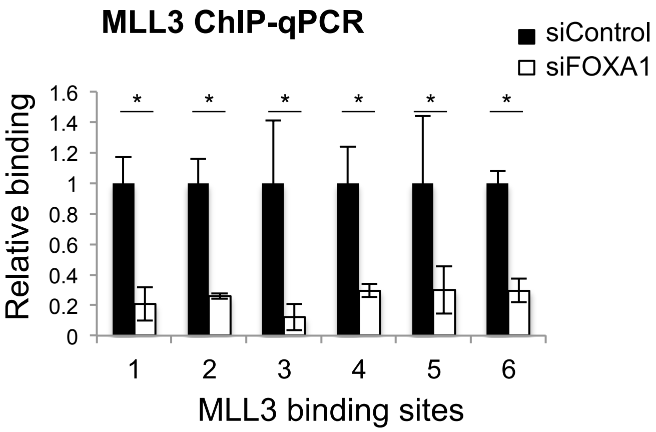

Supplementary Figure 2D. Genomic regions around the *TFF1*, *MYC* and *PGR* loci, showing the ER, MLL3, FOXA1, GRHL2 and histone mark signal. Also included is transcription signal (GRO-seq).

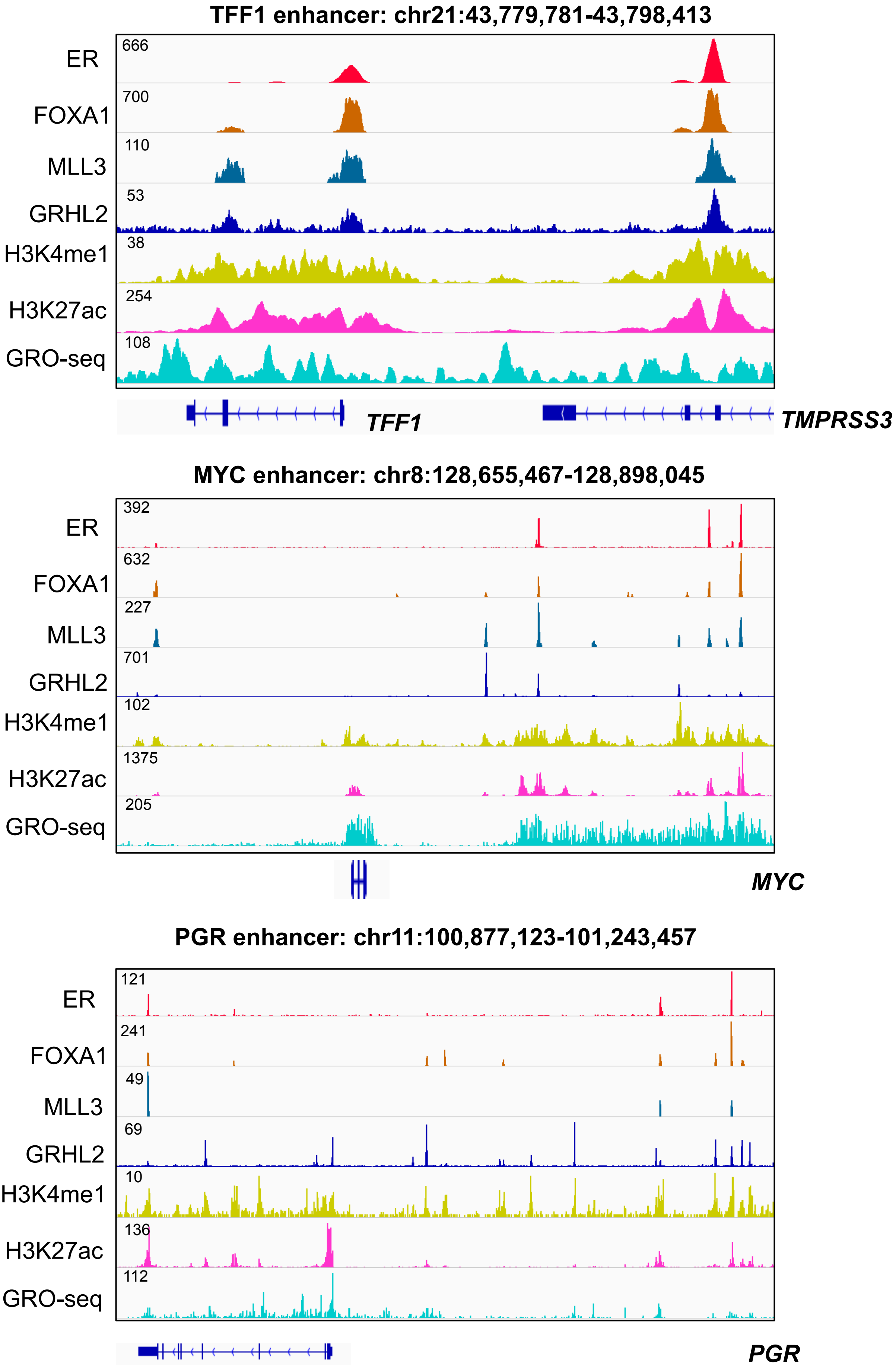

Supplementary Figure 2E. FOXA1 is required for H3K27Ac. MCF-7 cells were transfected with siControl or siFOXA1 and H3K27Ac or H3K27me3 ChIP was performed. qPCR was conducted on known FOXA1 binding enhancers.

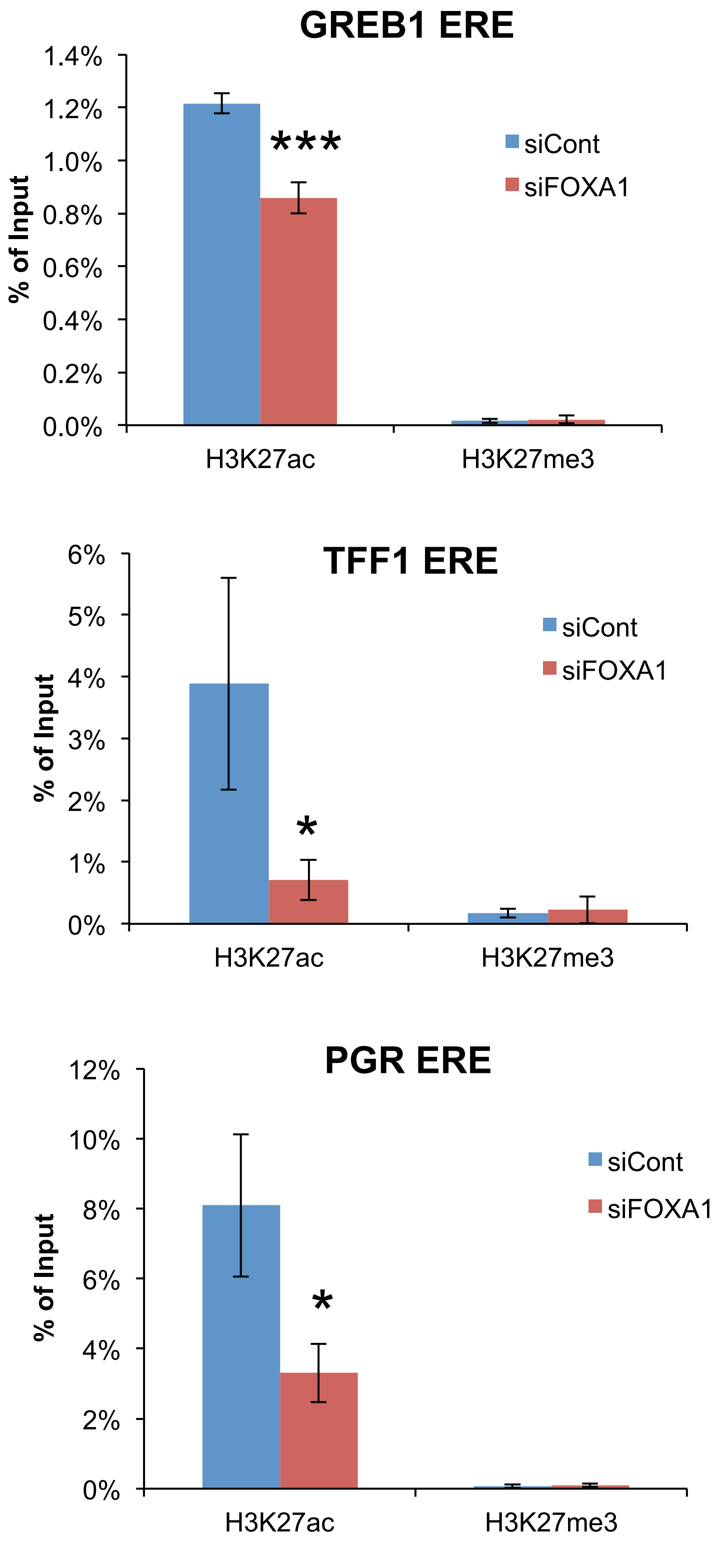

Supplementary Figure 2. (F) Effect of FOXA1 knockdown on H3K4me1 occupancy. (G) Effect of MLL3 knockdown on the estrogen-induced expression of GREB1. *p ≤ 0.05, ***p ≤ 0.001. (H) Genomic regions around the *GREB1* loci, showing the ER, MLL3, FOXA1, GRHL2 and histone mark signal. Also included is transcription signal (GRO-seq).

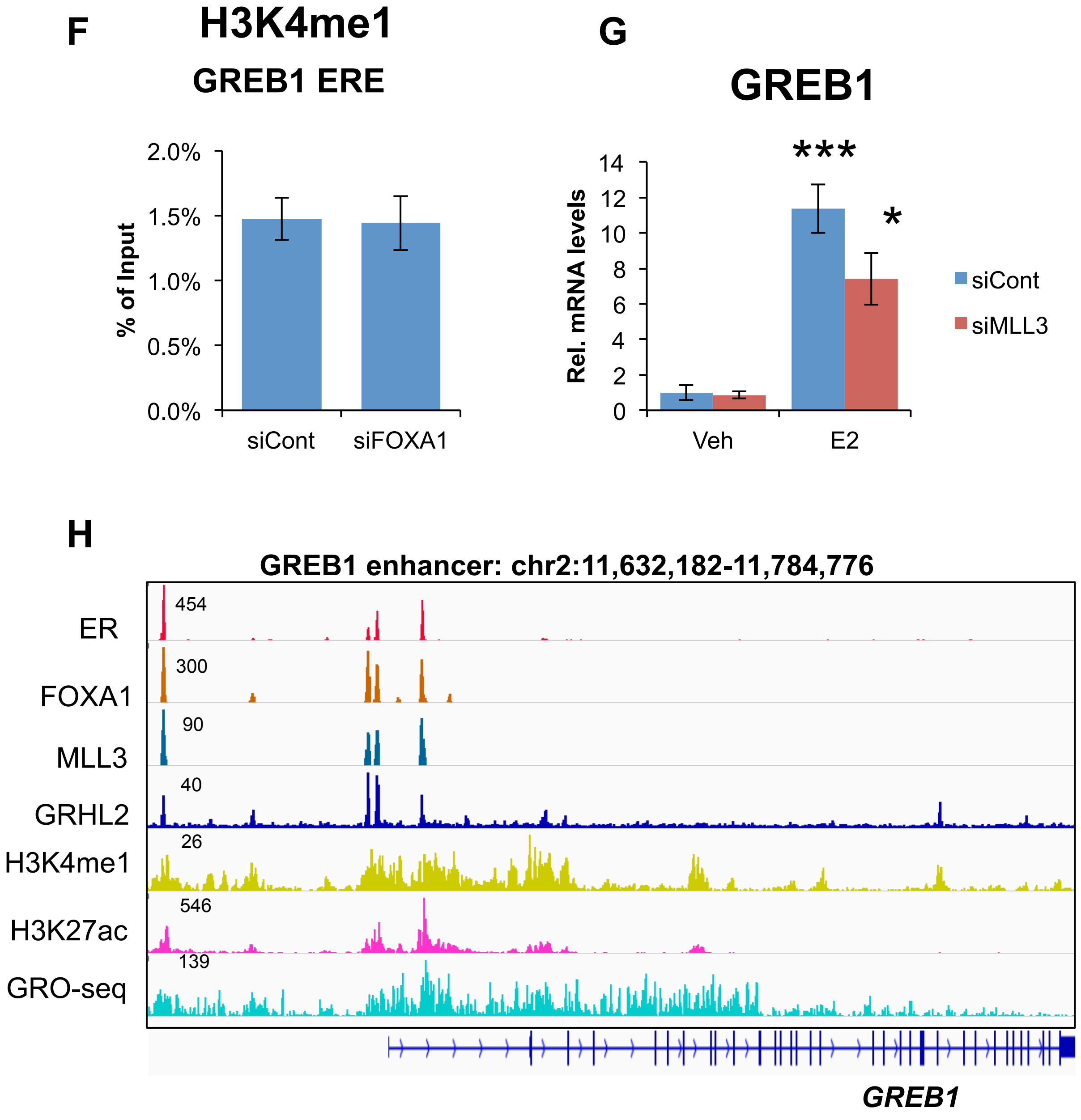

Supplementary Figure 3. Venn diagram showing proportion overlap between ChIP-seq peak sets for FOXA1, MLL3, ER and GRHL2. As expected from previous findings, ~50% of ER peaks overlap with FOXA1.

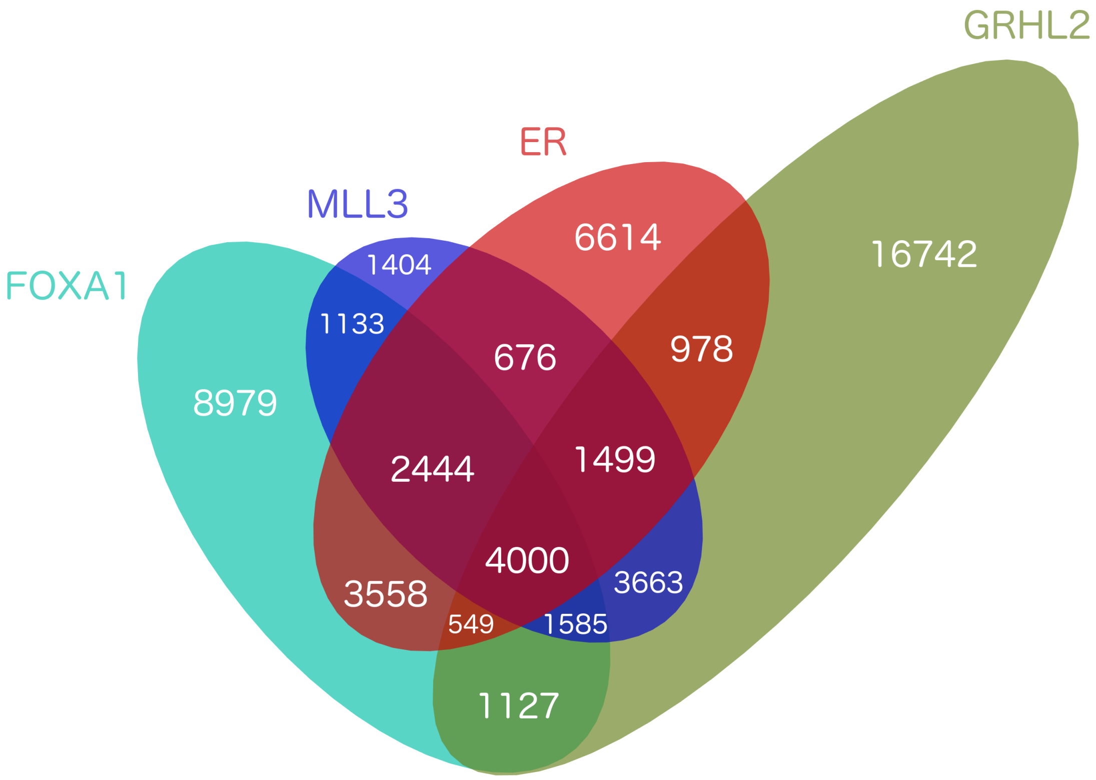

Supplement: Document S1. Figures S1–S3 [file mmc1.pdf]
